# Supplementary material for: Predicting the water film depth: A model based on the geometric features of road and capacity of drainage facilities
Source: PLoS One. 2021 Jul 2;16(7):e0252767. doi: 10.1371/journal.pone.0252767 (PMC8253438; doi:10.1371/journal.pone.0252767)
Supplement: S1 File — (PDF) [file pone.0252767.s001.pdf]

All the raw data used in this paper includes the depth of water film and rainfall intensity.

In 2019-2020, field measurements of water film depth in rainy days were conducted on several highways in Shaanxi Province, Shandong Province, and Zhejiang Province. Pictures of the field trials have been added to the manuscript. The length and width of the catchment should be determined in accordance with the actual resultant gradient of road.

(i)The depth of water film was measured on Taibai highway. The rainfall intensity is 1.2mm/h, the catchment length is 5m, the catchment width is 4m and the horizontal and longitudinal slope is 2%. Under this condition, we measured the depth of water film in 5 min, 10 min, 30 min and 60 min respectively, and took the average value as the depth of water film under the current rainfall intensity, as shown in Table 1.

Table 1 Measured data of the depth of water film

Number	Time(min)	Measured value(mm)	Average value(mm)
1	5	1.3	1.4
2	10	1.4	
3	30	1.4	
4	60	1.4	

(ii) Field observation experiment of rainfall intensity was carried out in Shaanxi Province, Shandong Province, and Zhejiang Province, as shown in Table 2.

Table 2 Measured data of rainfall intensity

experimental section	rainfall intensity (mm)			
	5 min	10 min	30 min	60 min
Taibai highway	0.1	0.3	0.6	1.2
Binlai expressway	0.2	0.5	1.6	3.5
Hangshaotai expressway	0.5	0.9	3.1	6.2
Hangshaotai expressway	2.1	4.0	9.8	14.9
Hangshaotai expressway	4.1	7.5	19.7	38.2
