# Supplementary material for: Predicting the water film depth: A model based on the geometric features of road and capacity of drainage facilities
Source: PLoS One. 2021 Jul 2;16(7):e0252767. doi: 10.1371/journal.pone.0252767 (PMC8253438; doi:10.1371/journal.pone.0252767)
Supplement: Attachment — Submitted filename: Response to Reviewers.doc [file pone.0252767.s002.doc]

# TO Reviewer #1:

We appreciate your time and help in reviewing our manuscript. Thank you very much for your affirmation of our work in this paper and many thanks for your detailed comments. We have revised the paper very carefully according to your suggestion, and all the modified contents have been marked in red font in the updated manuscript.

**Response:**

**1.In general, your English writing needs to improve. Make sure that readers can understand your paper without difficulties. Your abstract has so many grammar mistakes.**

**Response:**

Many thanks for your suggestion.

The English of the full manuscript has been made improvement in the following aspects:

1.We have improved the language expression logic to guide the readers better.

2.We have reorganized language expressions for the verbose passages to make it easier for readers to understand.

3.We have revised the grammar and typos in the original text.

4.We have enriched vocabulary expressions.

5.We have tried to avoid the use of first-person pronouns.

We reorganized and compiled the abstract as follows.

The depth of water film is a key variable to affect traffic safety under rainfall. Figure out the correlation among the depth of water film and the geometric features of road and drainage capacities of facilities is essential for road safety under a rainy day. First, the road geometry feature was classified into four types, and the facility drainage capacities were concerned from three aspects in this work. Further, the concept of short-time rainfall grade was proposed according to the results of the field test. Finally, the prediction model for the depth of water film based on the geometric features of road and drainage capacity of facilities with different rainfall intensities was conceived. Results show that the depth of water film increases with the increase in rainfall intensity in the case of keeping some parameters constant such as road alignment and materials. Under such circumstance that the drainage facilities on highway are in normal service, the error of the model for depth of water film is 0.15mm when compared with the data measured from the field test on rainy days. Compared with the existing model results, the difference is not significant. This prediction model can be used to evaluate the depth of water film for highway on rainy days. This prediction model can be used to evaluate the depth of water film for highway on rainy days, offering guidance on the measurement of road friction coefficient and the arrangement of security controls on rainy days.

**2.Literature Review (in your Introduction section) also needs to improve, please read more papers and learn how to write it, please figure out outstanding opinions.**

**Response:**

Many thanks for pointing out this issue.

We reorganized and compiled the Literature Review as follows.

Researches have shown that the wet and slippery road accompanied with the decrease of friction caused by rainfall is one of the key variables in traffic accidents [1-4]. According to the statistics from the Korean Transportation Safety Authority, although the total number of deaths due to the road traffic accidents decreased in 2013-2016 in Korea, the number of deaths related to rainfall increased from 430 in 2013 to 463 in 2016. Lee et al. concluded that rain and water depth factors and road factors were mutually correlated with the level of accident severity [1]. Research by Brodsky et al. showed that the accident rate on wet and slippery roads on rainy days was 2 to 3 times higher than that on dry roads on sunny days. Moreover, the longer stopping sight distance needed due to a reduction in friction between the tiers and the wet road surface might be more challenging on curves. In a 1980 study conducted by the National Transportation Safety Board, it was found that in the United States, the risk of fatal accidents on wet roads is 3.9 to 4.5 times that on dry roads [3]. Research by Qiu et al. showed that rainfall led to an average increase of 71% in traffic accident rates, which had a significant impact on traffic accidents [4]. Yu et al. found that the probability of a collision increased significantly for drivers traveling through downgrade steep segments under large precipitation conditions. The study also proved that the rain collision rate is related to different road sections and road geometric characteristics [5].To find out the causes of high incidence of road traffic accidents on rainy days, researchers focused on the relationship between the friction coefficient and the depth of water film, and reached the consensus that there was a direct correlation between the reduction of friction coefficient and the depth of water film on road surface [6-8]. Through indoor water spray experiments, Do et al. measured the friction coefficient under water depth (<1mm), and the friction force-water depth curve was conformed to the Stribeck curve [6]. A finite element simulation model was established to predict the friction coefficient under different water film depth, and found that the friction coefficient decreased with the increase of water film depth at the same driving speed [7].Using a dynamic friction coefficient tester, dynamic friction coefficient test experiments were carried out under different water film depth through artificial rainfall experiments and found that the decrease in friction coefficient is directly related to the depth of water film on the pavement [8]. The thicker the water film is, the worse the skid resistance is. Even worse, the hydroplaning phenomenon may occur, imposing severe impact on the driving safety under rainfall [9-11]. Hence, a prediction model for depth of water film is vital in determining the road friction coefficient and managing the security controls on rainy days. As is known, the highway traffic environment is complex in the rain, various geometric features of road and drainage capacities of facilities have different influences on the depth of water film, and so does the different rainfall intensities. Luo et al. used variance analysis and multi-factor treatment statistical methods, and concluded that the calculation of water film depth is affected by rainfall (rainfall intensity, duration) and pavement characteristics (cross slope, longitudinal slope, pavement material properties, texture, and penetration), and the flow path slope has a greater impact on surface drainage than the length of the flow path [12].Concerning such complex factors as aforementioned, numerous investigations on the depth of water film have been carried out, and different prediction models have been developed. However, most prediction models were established based on the in-door simulation experiments, which is ill-considered for the actual road circumstances and external environmental conditions such as wind, causing it being limited in practical applications. For example, based on the in-door rainfall simulation experiment, the UK Road Research Laboratory proposed the typical RRL model, i.e., the depth of rain water relating drainage length, rainfall intensity (5 minutes duration), and slope [13]. B. M. Gallaway et al developed the model of the effects of rainfall intensity, pavement cross slope, surface texture, and drainage length on water depth [14]. On the basis of Gallaway model, R. S. Huebner et al built up the PAVDRN computer model, which presented that rainfall intensity (5 minutes duration), pavement section geometry and structure accounted for the depth of water film [15]. Based on the classical RRL and Gallaway models, the water film depth regression model was calibrated according to the data from test by J. Luo et al [16-18]. With the Gallaway model framework, J. Chesterton et al gave a forecast of the depth of water film for New Zealand highway [19]. And using the collision data in Florida, W. Jayasooriya et al. evaluated the prediction precision of the Gallaway and PAVDRN models for the depth of water film and hydroplaning speed [20].

Clearly, the models mentioned above are in accordance with some specific test data in their experience. However, because of the three-dimensional structures of the actual roads, the combinations of road slope, superelevation and alignment are complex and variable to adapt to the terrain variation and engineering requirements. The drainage capacities of the drainage facilities will undergo some changes in their service lives. Taking such factors into consideration, the effects of such complex factors on the depth of pavement water film may not be illustrated clearly using the models available now. Therefore, the parameters cannot be modified according to the actual situation, imposing restrict on the fields of applications for them.

**3.Please clarify the innovations, and explain the difference between your work and others, high light your contributions. You can add these in the section of Introduction.**

**Response:**

Many thanks for pointing out this issue.

To explain the difference between our work and others, highlight our contributions, we added two main advantages of this study in the section of Introduction as follows.

To overcome the limitations of the aforementioned methods, and thus give an accurate prediction for the correlation between the depth of water film and the geometric features of road and drainage capacities of facilities, we proposed a prediction model for water film depth in theory. More specifically, the road geometry feature was considered from four types and the facility drainage capacities were concerned from three aspects in this work, and the rainfall intensity involved short time light rain, moderate rain, heavy rain, rainstorm, and heavy storm. And the prediction model for the depth of water film based on the geometric features of road and drainage capacity of facilities with different rainfall intensities was developed. The two main advantages of this paper are as follows: First, the study puts forward a classification of short-term rainfall. Second, the model established at the theoretical level clarifies the influence of road geometric characteristics and drainage capacity of drainage facilities on the depth of water film. The results can provide guidance on the measurement of road friction coefficient and the arrangement of security controls on rainy days.

**4. In section Rainfall, you give the equation, if this quote from others, please give the reference. Otherwise, please explain how you get this equation. The other equations also need explanations.**

**Response:**

Many thanks for pointing out this problem.

Formula 11 is derived from drainage design specifications (Ministry of communications of china, Design Specification for Highway Alignment, PRC Industry Standard JTG D20-2017). The reference has been added to the text, and the rest of the formulas are derived from the perspective of mathematical models. The formulas are explained in detail below.

The geometric models for water film depth with different road geometric characteristics are established based on the different combinations of cross slope and longitudinal slope, and the different convergence directions of slop flow. The amount of gathered water is the volume of the gathered water in the geometric model, and the water film depth is the depth in the geometric model. From the perspective of mathematical models, formulas are used in this paper to derive the volume and water film depth with different geometric models.

During rainfall, this work focuses on the situation of setting water retaining belts or edge stones, such as Shandong Binlai Expressway. Because of the different combinations of road alignment and the effect of road alignment, water retaining belt or edge stone on rainfall, the rainfall will be collected on the highway surface, forming slope flow and then being discharged from the drainage facilities. The amount of rainfall is equal to the volume of converged slope flow in unit time.

Formula 1:

In this work, the amount of rainfall is defined as the volume of rainwater accumulated on the water surface without evaporation, infiltration and runoff, which comes from the rainwater falling from the sky to the ground in the case of a certain catchment area, sharing the unit with m3/h. The amount of rainfall is related to the rainfall intensity and catchment area, which can be described as the following equation:

$Q=I_{R}F\times10^{\text{-}3}=I_{R}LW\times10^{\text{-}3}$ (1)

where the *Q*, *I_R_*, *F*, *L*, and *W* represent the amount of rainfall (m^3^/h), rainfall intensity (mm/h), catchment area (m^2^), catchment length (m), and catchment width (m), respectively.

Formula 2-4:

Simply for ease of calculation, assuming that a cylinder with the bottom radius R is cut by a plane at an angle β. Therefore, the intercepted cylinder is an inclined cylinder. And a bow height H is formed at the bottom, as shown in Fig 5. And the bottom of the cylinder is presented in Fig 6. It can be concluded that the depth of the road surface water film is the bow height H, and the volume of the accumulated water is equivalent to the volume of the scalene cylinder cut by the cross section.

**Fig 5. Geometric diagram of a scalene cylinder cut by cross section.**

**Fig 6. Geometric diagram of the bottom of cylinder.**

When the section is cut by a cross section plane perpendicular to x axis, each section sharing with a right triangle can be obtained, and the function of cross section area A(x) can be described as follows:

$A(x)=\frac{1}{2}BD\cdot BF\text{=}\frac{1}{2}BD^{2}\tan\beta$ (2)

$BD=BG-DG=\sqrt{R^{2}-x^{2}}-OC=\sqrt{R^{2}-x^{2}}-(R-H)$ (3)

Therefore, $A(x)=\frac{1}{2}\left[\sqrt{R^{2}-x^{2}}-(R-H)\right]^{2}\tan\beta$ (4)

Where the $A(x)$, $R$, $H$, $x$, and$\beta$ are the area of cross section (m^2^), radius of the vertical curve (m), depth of water film (m), the vertical distance between cross section BDF and the y-axis (m), and angle of cross section CEI to the bottom (°), respectively.

Formula 5:

Formula 5 is the mathematical derivation process of the volume of an inclined cylinder, and in this paper represents the volume of water accumulated in model A.

The volume of the accumulated water on road surface, i.e., the volume of the inclined cylinder cut by the cross section $V_{j1}(H)$, can be derived as shown in the following:

$\begin{matrix} V_{j1}(H)=2\int_{0}^{\sqrt{R^{2}-(R-H)^{2}}} \frac{1}{2}\left[\sqrt{R^{2}-x^{2}}-(R-H)\right]^{2}\tan\beta dx \\ =\left\{\sqrt{R^{2}-(R-H)^{2}}\left[\frac{2}{3}R^{2}+\frac{1}{3}(R-H)^{2}\right]-R^{2}(R-H)\arcsin\frac{\sqrt{R^{2}-(R-H)^{2}}}{R}\right\}\tan\beta\\ =\left\{\sqrt{R^{2}-(R-H)^{2}}\left[\frac{2}{3}R^{2}+\frac{1}{3}(R-H)^{2}\right]-R^{2}(R-H)\arcsin\frac{\sqrt{R^{2}-(R-H)^{2}}}{R}\right\}\frac{1}{i_{h}} \end{matrix}$ (5)

where the $V_{j1}(H)$, *R*, *H*, and *i_h_* stand for the volume of the accumulated water on road surface (m^3^) with model A, radius of the vertical curve (m), the depth of water film (m), and the horizontal slope, respectively.

Formula 6:

Formula 6 is the mathematical derivation process of the volume of a triangular prism, and in this paper represents the volume of water accumulated in model B.

Formula 7-9:

Formula 7-9 calculate the accumulated water volume of Model C from the perspective of mathematical derivation

The shadow area S is equal to the area difference between sector and triangle:

$S=2\left[\frac{1}{2}\alpha R^{2}-\frac{1}{2}(R-H)R\sin\alpha\right]$ （7）

Where the *S*，$\alpha$，*R*，and *H* are the shadow area (m^2^), central angle of the sector (rad), radius of the vertical curve (m), and depth of water film (m), respectively.

$\alpha\text{=arccos}\frac{R-H}{R}$ (8)

$V_{j3}(H)=SW=\left[R^{2}\arccos\frac{R-H}{R}-(R-H)\sqrt{R^{2}-(R-H)^{2}}\right]W$ (9)

where the *V_j3_(H)*, *R*, *W*, and *H* are the volume of the accumulated water on road surface (m^3^) with model C, *radius* of the vertical curve (m), catchment width (m), and the depth of water film, respectively.

Formula 10:

Formula 10 is the mathematical derivation process of the volume of a triangular prism, and in this paper represents the volume of water accumulated in model D.

Formula 11:

Formula 11 is derived from drainage design specifications (Ministry of communications of china, Design Specification for Highway Alignment, PRC Industry Standard JTG D20-2017). The reference has been added to the text.

Formula 12-16:

The depth of water film is related to the rainfall intensity and the drainage capacities of facilities. Taking the road with drainage facilities or not and the capacities of drainage facilities as measures, the calculation of the depth of water film(Formula 12-16) can be conducted from three situations, which includes the road with drainage facilities and in normal use, the road with drainage facilities of poor capacities, and the road without drainage capacities.

The depth of the water film is related to rainfall, geometric model of road water accumulation, and drainage volume. Drainage is related to the capacity of road drainage facilities. Taking the road with drainage facilities or not and the capacities of drainage facilities as measures, the calculation of the depth of water film can be conducted from three situations, which includes the road with drainage facilities and in normal use, the road with drainage facilities of poor capacities, and the road without drainage capacities.

**Road with Drainage Facilities and in Normal Use**

When the road is equipped with drainage facilities and the facilities are in their normal use, the water displacement from drainage facilities *Q_c_* can be greater than or equal to the amount of rainfall *Q*. The rainfall can be completely discharged and there will be no water accumulation on the road surface. Thus, the *Q* is equal to *Q_c_*. During rainfall, the depth of water at the flow section is the real-time depth of water film of pavement. The model of depth of water film can be expressed as follows:

$3600Q_{c}=Q$ (12)

Where *Q_c_*. and *Q* refer to the amount of water displacement from drainage facilities and the amount of rainfall, respectively.

$H=0.005\left(\frac{I_{R}LWi_{h}n}{I^{\frac{1}{2}}}\right)^{\frac{3}{8}}$ (13)

Where $H$，$I_{R}$，$L$，$W$，$i_{h}$，$n$，and $I$ stand for the water film depth (m), rainfall intensity (mm/h), catchment length (m), catchment width (m), cross slope of flow section, roughness coefficient of trench wall or pipe wall (0.013 for asphalt pavement), and hydraulic gradient, i.e., longitudinal gradient of road.

**Road with Drainage Facilities of Poor Capacities**

When the road has drainage facilities but the facilities takes on poor drainage capacities, the amount of rainfall *Q* will be greater than water displacement from drainage facilities *Q_c_*. Thus, the rainfall cannot be completely discharged, causing water accumulating on the road. The designed water displacement of drainage facility is marked as *Q_cmax_*, and the *Q_cmax_* is related to the setting of highway drainage facilities. The drainage capacities are different because of different timely maintenance of drainage facilities. To be clear of the change degree of drainage capacity of the facilities, the performance evolution coefficient *a* is introduced, and the value of *a* is 1 for the new drainage facilities. In general, the value of *a* is less than or equal to 1, which is relevant to the timeliness of maintenance of the drainage facilities. The actual drainage capacity of facility labeled as .

In this case, the volume of the accumulated water can be described as follows:

(14)

where *V*, *Q*, *a*, and *Q_cmax_*, represent the volume of the accumulated water on road(m^3^), amount of rainfall (m^3^/h), performance evolution coefficient of drainage facilities, and theoretical amount of water displacement of drainage facilities(m^3^/s), respectively.

In addition, different road water accumulation models can be obtained owing to various road alignments and combinations, and accordingly different depths of water film can be observed. Therefore, the model of depth of water film in the case of the road with drainage facilities of poor capacities can be expressed as follows:

(15)

where the *V_ji_(H), Q*, *a*, and *Q_cmax_*, are the corresponding amount of the accumulated water to different road geometry models (m^3^/h, number of 1, 2, 3, or 4 for variable *i*), amount of rainfall (m^3^/h), performance evolution coefficient of drainage facilities, and the designed amount of water displacement of drainage facilities (m^3^/h), respectively.

**Road without Drainage Capacities**

With the increase of the service life of highway, changes have occurred in the geometric alignment indexes of road and the road drainage capacity, giving rise to the road section with no drainage capacities, which remains a hidden trouble for driving safety during rainfall. In this case, all the rainfall will be collected on the road surface. Rainfall is equal to the amount of the accumulated water and the corresponding depth of water film model can be described as follows:

$V_{ji}(H)=Q$ (16)

where the *V_ji_(H)* is the corresponding amount of the accumulated water to different road geometry models (m^3^/h), and the variable *i* is a number of 1, 2, 3, or 4.

**5.You have given several assumptions in this paper, please explain why this is reasonable. Also the explanations of the four models you choose in this paper.**

**Response:**

Many thanks for your question. We elaborated this issue as follows:

When the water is gathered on the surface, there will be water film with a certain of depth, which is the basic idea of the water film depth prediction model. The amount of surface gathered water is defined as the amount of rainfall subtracts the amount of drainage, which means that the volume of surface gathered water in unit time is the difference between the volume of rainfall and drainage. Despite the same volume of gathered water, the different road geometry features and convergence directions of flow cause different depths of water film. The amount of rainfall is related to the rainfall intensity, and the amount of drainage is bound up with the facility drainage capacities. Therefore, the prediction model for water film depth is proposed here based on the geometric features of road and drainage capacity of facilities with different rainfall intensities.

For the purpose of field verification and model derivation, the pavement water film depth in rainy days mentioned here is defined as the depth of water film under short-time (1h) rainfall. Compared with the highway without water retaining edge stone such as Hangshaotai Expressway, the rainfall will converged on the road surface, forming slop flow and then being discharged from the drainage facilities for the highway with water retaining belt or edge stone. Therefore, this work focuses on the depth of water film at the outside edge of hard shoulder in the case of setting water retaining belts or edge stones. The road surface water film depth is related to the amount of accumulated water, which refers to the difference between the amount of rainfall and the water displacement of drainage facilities. Albeit with the same water accumulation, various road alignments and combinations result in different depths of water film. Hence, the construction of the model about the depth of water film includes the calculation of rainfall, construction of road water accumulation model, calculation of water displacement of drainage facilities, and derivation of formulas about road surface water film depth.

A prediction model for water film depth is proposed theoretically in this paper. And to meet the requirements of topographic changes and engineering, the effects of rainfall evaporation, infiltration and runoff, and the pavement material properties are ignored.

The reason for water gathering on surface locates in the road section with poor drainage. Despite the same volume of gathered water, the different road geometry features and convergence directions of flow cause different depths of water film. According to the values of horizontal and longitudinal gradients, there are 4 types for the road section with poor drainage, following with 4 geometric models for road water accumulation.

The geometric models for water film depth with different road geometric characteristics are established based on the different combinations of cross slope and longitudinal slope, and the different convergence directions of slop flow. The amount of gathered water is the volume of the gathered water in the geometric model, and the water film depth is the depth in the geometric model.

**6.You may give a case study to show your model, and some GIS figures will be more visible for showing the depth of water on roads.**

**Response:**

Thank you very much for your suggestion and expanding our knowledge.

Due to time issues, we chose the familiar UC-Win/Road simulation software, which is a 3D virtual reality tool widely used on highways. We wonder if it can meet your expectations. The UC-Win/Road simulation software was used to build 3 highway models of different geometric characteristics. In order to better describe the model, we added a few figures generated by UC-Win/Road simulation software and AutoCAD, as shown below.

For the road section with good horizontal drainage and poor longitudinal drainage (Model B), the depth of water film increases with the increase in rainfall intensity when the catchment length, catchment width and longitudinal gradient remain unchanged. Fig 10 shows the depth of water film of road with rainfall lasting for 1h. Given the catchment length of 25m, catchment width of 11.25m, longitudinal gradient of 0.3% and rainfall intensity of 46 mm/h under the short-time heavy storm, the corresponding depth of water film is 83mm, as shown in Figure 12.

**Fig 12. Schematic diagram of the depth of water film (Model B).**

For the road section with poor drainage in both horizontal and longitudinal slope (Model C), when keeping the radius of vertical curve, catchment length and catchment width unchanged, the depth of water film will increase as rainfall intensity increases. As shown in Fig 10, the depth of water film is 29.2mm in the case of water catchment length of 25m, catchment width of 11.25m, radius of vertical curve of 15000m and rainfall intensity of 46mm/h under the short-time heavy storm, as shown in Figure 13.

**Fig 13. Schematic diagram of the depth of water film (Model C).**

For the road section with poor drainage in cross slope and good drainage in longitudinal slope (Model D), when no changes occur in the catchment length, catchment width and longitudinal gradient, the depth of water film will increase with the increase in rainfall intensity. As shown in Fig 10, under the condition of short time heavy storm, a depth of water film of 55.8mm can be obtained corresponding to the situation with the catchment length of 25m, catchment width of 11.25m, horizontal gradient of 0.3%, and rainfall intensity of 46mm/h, as shown in Figure 14.

**Fig 14. Schematic diagram of the depth of water film (Model D).**

# To Reviewer #2:

Thank you very much for the detailed comments. We appreciate your time and help in reviewing our manuscript, and the insightful comments you provided that have helped significantly improve the quality of this study. We have revised the paper very carefully according to your suggestions, and detailed explanations of all the issues are as follows.

**Response:**

**1.Abstract: Setting a need for research in the depth of water film shall have background data. If data on accidents happening due to poor road conditions is presented then that will help in understanding the gravity of situation and need of the study.**

**Response:**

Many thanks for pointing out this problem.

To better understand research need, background data has been added in Introduction section.

Researches have shown that the wet and slippery road accompanied with the decrease of friction caused by rainfall is one of the key variables in traffic accidents [1-4]. According to the statistics from the Korean Transportation Safety Authority, although the total number of deaths due to the road traffic accidents decreased in 2013-2016 in Korea, the number of deaths related to rainfall increased from 430 in 2013 to 463 in 2016. Lee et al. concluded that rain and water depth factors and road factors were mutually correlated with the level of accident severity [1]. Research by Brodsky et al. showed that the accident rate on wet and slippery roads on rainy days was 2 to 3 times higher than that on dry roads on sunny days. Moreover, the longer stopping sight distance needed due to a reduction in friction between the tiers and the wet road surface might be more challenging on curves. In a 1980 study conducted by the National Transportation Safety Board, it was found that in the United States, the risk of fatal accidents on wet roads is 3.9 to 4.5 times that on dry roads [3]. Research by Qiu et al. showed that rainfall led to an average increase of 71% in traffic accident rates, which had a significant impact on traffic accidents [4]. Yu et al. found that the probability of a collision increased significantly for drivers traveling through downgrade steep segments under large precipitation conditions. The study also proved that the rain collision rate is related to different road sections and road geometric characteristics [5].

**2.If there is a relationship between the depth of water film and the driving risk level, it is better to put forward the driving risk level according to the prediction results of the model, so as to put forward the safety control measures in rainy days.**

**Response:**

Thank you very much for your suggestion and expanding my knowledge.

Researchers conducted works about the water film depth and driving risk. Typical representatives include the researches of Ahmed, Do M.-T., Peng J. [R1-R3]. The water film depth has impact on road friction coefficient, which affects the lateral driving stability. Too small friction coefficient may cause slippage and overturning of driving, and increase the braking distance when braking, causing safe velocity lower than the standard driving speed and raising the driving risk. However, a constant friction coefficient is adopted for safe velocity calculation in current specifications such as AASHTO, which loses sight of the effect of pavement water film depth. Unfortunately, we do not discuss such a point in this paper because of the workload. And we will make a further study it in next steps. Thanks again for your suggestions.

References cited in the response letter here for your convenience:

[R1] Mohamed M. Ahmed, Ghasemzadeh A . The impacts of heavy rain on speed and headway Behaviors: An investigation using the SHRP2 naturalistic driving study data[J]. Transportation Research Part C: Emerging Technologies, 2018, 91:371-384.

[R2] Do M.-T., Cerezo V., Beautru Y., Kane M. Modeling of the connection road surface microtexture/water depth/friction. Wear, 2013; 302(1-2): 1426-1435.

[R3] Peng J., Chu L., Fwa T. F. Determination of safe vehicle speeds on wet horizontal pavement curves. Road Materials and Pavement Design. 2020. DOI: 10.1080/14680629.2020.1772350.

**3.I think F in formula (1) is redundant.**

**Response:**

Many thanks for pointing out this problem. We reinterpreted the F (catchment area) as follows.

The pavement water film depth in rainy days is defined as the depth of water film at the outside edge of hard shoulder under short-time (1h) rainfall. During rainfall, this work focuses on the situation of setting water retaining belts or edge stones, such as Shandong Binlai Expressway. Because of the different combinations of road alignment and the effect of road alignment, water retaining belt or edge stone on rainfall, the rainfall will be collected on the highway surface, forming slope flow and then being discharged from the drainage facilities, distance between which is usually set to be 25~50m. Therefore, the effect of catchment area (F) is involved in the calculation in this work.

When the water is gathered on the surface, there will be water film with a certain of depth, which is the basic idea of the water film depth prediction model. The amount of surface gathered water is defined as the amount of rainfall subtracts the amount of drainage, which means that the volume of surface gathered water in unit time is the difference between the volume of rainfall and drainage. Despite the same volume of gathered water, the different road geometry features and convergence directions of flow cause different depths of water film. The amount of rainfall is related to the rainfall intensity, and the amount of drainage is bound up with the facility drainage capacities. Therefore, the prediction model for water film depth is proposed here based on the geometric features of road and drainage capacity of facilities with different rainfall intensities.

In this work, the water film depth refers to that on the highway with water retaining belt or edge stone. Typical representative for the case of overflow drainage, i.e., no water retaining edge stones on pavement, is the Hangzhou Shaoxing Taizhou Expressway. In the case of the pavement with good drainage and ignoring the effects of such factors as pavement texture, material properties and converge flow, it can be considered roughly as that the real-time water film depth during rainfall is equal to the rainfall intensity.

**4.Line 208, the format of “(JTGT_D33-2012)” is incorrect.**

**Response:**

Many thanks for pointing out this issue.

We have revised the format as follows: “(JTG/T D33-2012)”.

# To Reviewer #3:

We appreciate your time and help in reviewing our manuscript. Thank you very much for your affirmation of our work in this paper and many thanks for your detailed comments. We have revised the paper very carefully according to your suggestion, and all the modified contents have been marked in red font in the updated manuscript.

**Response:**

**1.Lin 215. “Calculation of the Depth of Water Film Depth” should be “Calculation of the Water Film Depth”.**

**Response:**

Many thanks for pointing out this issue.

We have revised the heading as follows: “Calculation of the Water Film Depth”.

**2.Whether the formula or model involved in the paper is based on the others research results? are they all derived by yourself?**

**Response:**

Formula 11 is derived from drainage design specifications (Ministry of communications of china, Design Specification for Highway Alignment, PRC Industry Standard JTG D20-2017). The reference has been added to the text, and the rest of the formulas and models are derived from the perspective of mathematical models. The formulas and models are explained in detail below.

The geometric models for water film depth with different road geometric characteristics are established based on the different combinations of cross slope and longitudinal slope, and the different convergence directions of slop flow. The amount of gathered water is the volume of the gathered water in the geometric model, and the water film depth is the depth in the geometric model. From the perspective of mathematical models, formulas are used in this paper to derive the volume and water film depth with different geometric models.

During rainfall, this work focuses on the situation of setting water retaining belts or edge stones, such as Shandong Binlai Expressway. Because of the different combinations of road alignment and the effect of road alignment, water retaining belt or edge stone on rainfall, the rainfall will be collected on the highway surface, forming slope flow and then being discharged from the drainage facilities. The amount of rainfall is equal to the volume of converged slope flow in unit time.

Formula 1:

In this work, the amount of rainfall is defined as the volume of rainwater accumulated on the water surface without evaporation, infiltration and runoff, which comes from the rainwater falling from the sky to the ground in the case of a certain catchment area, sharing the unit with m3/h. The amount of rainfall is related to the rainfall intensity and catchment area, which can be described as the following equation:

$Q=I_{R}F\times10^{\text{-}3}=I_{R}LW\times10^{\text{-}3}$ (1)

where the *Q*, *I_R_*, *F*, *L*, and *W* represent the amount of rainfall (m^3^/h), rainfall intensity (mm/h), catchment area (m^2^), catchment length (m), and catchment width (m), respectively.

Formula 2-4:

Simply for ease of calculation, assuming that a cylinder with the bottom radius R is cut by a plane at an angle β. Therefore, the intercepted cylinder is an inclined cylinder. And a bow height H is formed at the bottom, as shown in Fig 5. And the bottom of the cylinder is presented in Fig 6. It can be concluded that the depth of the road surface water film is the bow height H, and the volume of the accumulated water is equivalent to the volume of the scalene cylinder cut by the cross section.

**Fig 5. Geometric diagram of a scalene cylinder cut by cross section.**

**Fig 6. Geometric diagram of the bottom of cylinder.**

When the section is cut by a cross section plane perpendicular to x axis, each section sharing with a right triangle can be obtained, and the function of cross section area A(x) can be described as follows:

$A(x)=\frac{1}{2}BD\cdot BF\text{=}\frac{1}{2}BD^{2}\tan\beta$ (2)

$BD=BG-DG=\sqrt{R^{2}-x^{2}}-OC=\sqrt{R^{2}-x^{2}}-(R-H)$ (3)

Therefore, $A(x)=\frac{1}{2}\left[\sqrt{R^{2}-x^{2}}-(R-H)\right]^{2}\tan\beta$ (4)

Where the $A(x)$, $R$, $H$, $x$, and$\beta$ are the area of cross section (m^2^), radius of the vertical curve (m), depth of water film (m), the vertical distance between cross section BDF and the y-axis (m), and angle of cross section CEI to the bottom (°), respectively.

Formula 5:

Formula 5 is the mathematical derivation process of the volume of an inclined cylinder, and in this paper represents the volume of water accumulated in model A.

The volume of the accumulated water on road surface, i.e., the volume of the inclined cylinder cut by the cross section $V_{j1}(H)$, can be derived as shown in the following:

$\begin{matrix} V_{j1}(H)=2\int_{0}^{\sqrt{R^{2}-(R-H)^{2}}} \frac{1}{2}\left[\sqrt{R^{2}-x^{2}}-(R-H)\right]^{2}\tan\beta dx \\ =\left\{\sqrt{R^{2}-(R-H)^{2}}\left[\frac{2}{3}R^{2}+\frac{1}{3}(R-H)^{2}\right]-R^{2}(R-H)\arcsin\frac{\sqrt{R^{2}-(R-H)^{2}}}{R}\right\}\tan\beta\\ =\left\{\sqrt{R^{2}-(R-H)^{2}}\left[\frac{2}{3}R^{2}+\frac{1}{3}(R-H)^{2}\right]-R^{2}(R-H)\arcsin\frac{\sqrt{R^{2}-(R-H)^{2}}}{R}\right\}\frac{1}{i_{h}} \end{matrix}$ (5)

where the $V_{j1}(H)$, *R*, *H*, and *i_h_* stand for the volume of the accumulated water on road surface (m^3^) with model A, radius of the vertical curve (m), the depth of water film (m), and the horizontal slope, respectively.

Formula 6:

Formula 6 is the mathematical derivation process of the volume of a triangular prism, and in this paper represents the volume of water accumulated in model B.

Formula 7-9:

Formula 7-9 calculate the accumulated water volume of Model C from the perspective of mathematical derivation

The shadow area S is equal to the area difference between sector and triangle:

$S=2\left[\frac{1}{2}\alpha R^{2}-\frac{1}{2}(R-H)R\sin\alpha\right]$ （7）

Where the *S*，$\alpha$，*R*，and *H* are the shadow area (m^2^), central angle of the sector (rad), radius of the vertical curve (m), and depth of water film (m), respectively.

$\alpha\text{=arccos}\frac{R-H}{R}$ (8)

$V_{j3}(H)=SW=\left[R^{2}\arccos\frac{R-H}{R}-(R-H)\sqrt{R^{2}-(R-H)^{2}}\right]W$ (9)

where the *V_j3_(H)*, *R*, *W*, and *H* are the volume of the accumulated water on road surface (m^3^) with model C, *radius* of the vertical curve (m), catchment width (m), and the depth of water film, respectively.

Formula 10:

Formula 10 is the mathematical derivation process of the volume of a triangular prism, and in this paper represents the volume of water accumulated in model D.

Formula 11:

Formula 11 is derived from drainage design specifications (Ministry of communications of china, Design Specification for Highway Alignment, PRC Industry Standard JTG D20-2017). The reference has been added to the text.

Formula 12-16:

The depth of water film is related to the rainfall intensity and the drainage capacities of facilities. Taking the road with drainage facilities or not and the capacities of drainage facilities as measures, the calculation of the depth of water film(Formula 12-16) can be conducted from three situations, which includes the road with drainage facilities and in normal use, the road with drainage facilities of poor capacities, and the road without drainage capacities.

The depth of the water film is related to rainfall, geometric model of road water accumulation, and drainage volume. Drainage is related to the capacity of road drainage facilities. Taking the road with drainage facilities or not and the capacities of drainage facilities as measures, the calculation of the depth of water film can be conducted from three situations, which includes the road with drainage facilities and in normal use, the road with drainage facilities of poor capacities, and the road without drainage capacities.

**Road with Drainage Facilities and in Normal Use**

When the road is equipped with drainage facilities and the facilities are in their normal use, the water displacement from drainage facilities *Q_c_* can be greater than or equal to the amount of rainfall *Q*. The rainfall can be completely discharged and there will be no water accumulation on the road surface. Thus, the *Q* is equal to *Q_c_*. During rainfall, the depth of water at the flow section is the real-time depth of water film of pavement. The model of depth of water film can be expressed as follows:

$3600Q_{c}=Q$ (12)

Where *Q_c_*. and *Q* refer to the amount of water displacement from drainage facilities and the amount of rainfall, respectively.

$H=0.005\left(\frac{I_{R}LWi_{h}n}{I^{\frac{1}{2}}}\right)^{\frac{3}{8}}$ (13)

Where $H$，$I_{R}$，$L$，$W$，$i_{h}$，$n$，and $I$ stand for the water film depth (m), rainfall intensity (mm/h), catchment length (m), catchment width (m), cross slope of flow section, roughness coefficient of trench wall or pipe wall (0.013 for asphalt pavement), and hydraulic gradient, i.e., longitudinal gradient of road.

**Road with Drainage Facilities of Poor Capacities**

When the road has drainage facilities but the facilities takes on poor drainage capacities, the amount of rainfall *Q* will be greater than water displacement from drainage facilities *Q_c_*. Thus, the rainfall cannot be completely discharged, causing water accumulating on the road. The designed water displacement of drainage facility is marked as *Q_cmax_*, and the *Q_cmax_* is related to the setting of highway drainage facilities. The drainage capacities are different because of different timely maintenance of drainage facilities. To be clear of the change degree of drainage capacity of the facilities, the performance evolution coefficient *a* is introduced, and the value of *a* is 1 for the new drainage facilities. In general, the value of *a* is less than or equal to 1, which is relevant to the timeliness of maintenance of the drainage facilities. The actual drainage capacity of facility labeled as .

In this case, the volume of the accumulated water can be described as follows:

(14)

where *V*, *Q*, *a*, and *Q_cmax_*, represent the volume of the accumulated water on road(m^3^), amount of rainfall (m^3^/h), performance evolution coefficient of drainage facilities, and theoretical amount of water displacement of drainage facilities(m^3^/s), respectively.

In addition, different road water accumulation models can be obtained owing to various road alignments and combinations, and accordingly different depths of water film can be observed. Therefore, the model of depth of water film in the case of the road with drainage facilities of poor capacities can be expressed as follows:

(15)

where the *V_ji_(H), Q*, *a*, and *Q_cmax_*, are the corresponding amount of the accumulated water to different road geometry models (m^3^/h, number of 1, 2, 3, or 4 for variable *i*), amount of rainfall (m^3^/h), performance evolution coefficient of drainage facilities, and the designed amount of water displacement of drainage facilities (m^3^/h), respectively.

**Road without Drainage Capacities**

With the increase of the service life of highway, changes have occurred in the geometric alignment indexes of road and the road drainage capacity, giving rise to the road section with no drainage capacities, which remains a hidden trouble for driving safety during rainfall. In this case, all the rainfall will be collected on the road surface. Rainfall is equal to the amount of the accumulated water and the corresponding depth of water film model can be described as follows:

$V_{ji}(H)=Q$ (16)

where the *V_ji_(H)* is the corresponding amount of the accumulated water to different road geometry models (m^3^/h), and the variable *i* is a number of 1, 2, 3, or 4.

**3.The model in this paper does not take pavement materials (e.g. bituminous or cement) into account, which can be discussed in future research work, such as adding correction parameters to improve the model.**

**Response:**

Thank you very much for your suggestion and expanding our knowledge.

Due to the workload, the influence of pavement materials is not considered in the model. In this work, the amount of rainfall is defined as the volume of rainwater accumulated on the water surface without evaporation, infiltration and runoff, which comes from the rainwater falling from the sky to the ground in the case of a certain catchment area. However, it could not be ignored that the pavement texture and material properties have a great influence on the water film depth. This is also the work to be studied in the future.

**4.As you mentioned, the grade of short time rainfall is proposed, but where it is, I have not found, the specific standard should be given.**

**Response:**

Many thanks for your suggestion. The grade of short time rainfall is presented in Table 3 in Results.

Based on the field measurement of the rainfall duration of several groups within 60min, the grade of short time rainfall is proposed, as shown in Table 5. By comparing with the rainfall intensity levels published by the local meteorological bureau on the same day, it can be found that the short-term rainfall levels are consistent with the official data, which can be used to provide a theoretical basis for better linking the rainfall levels with the transportation industry in the future.

**Table5. The grade of short time rainfall**

| **grade** | **rainfall intensity (mm)** | | | |
| --- | --- | --- | --- | --- |
|  | **5 min** | **10 min** | **30 min** | **60 min** |
| Short time light rain | ＜0.2 | ＜0.3 | ＜0.7 | ＜2.0 |
| Short time moderate rain | 0.2~0.5 | 0.3~0.8 | 0.7~1.8 | 2.0~4.0 |
| Short time heavy rain | 0.5~1.2 | 0.8~2.0 | 1.8~4.0 | 4.0~8.0 |
| Short time rainstorm | 1.2~4.0 | 2.0~6.0 | 4.0~12.0 | 8.0~20.0 |
| Short time heavy storm | 4.0~10.0 | 6.0~15.0 | 12.0~30.0 | 20.0~45.0 |
