# Supplementary material for: Predicting the water film depth: A model based on the geometric features of road and capacity of drainage facilities
Source: PLoS One. 2021 Jul 2;16(7):e0252767. doi: 10.1371/journal.pone.0252767 (PMC8253438; doi:10.1371/journal.pone.0252767)
Supplement: Attachment — Submitted filename: Response to Reviewers.doc [file pone.0252767.s003.doc]

# TO Reviewer #1:

We appreciate your time and help in reviewing our manuscript. Thank you very much for your affirmation of our work in this paper and many thanks for your detailed comments. We have revised the paper very carefully according to your suggestion, and all the modified contents have been marked in red font in the updated manuscript.

**Response:**

**1. Your English writing needs to improve. The Abstract and Literature Review have been improved, while they need further improvement , as well as other sections. Don't repeat same sentence:“This prediction model can be used to evaluate the depth of water film for highway on rainy days. This prediction model can be used to evaluate the depth of water film for highway on rainy days, offering guidance on the measurement of road friction coefficient and the arrangement of security controls on rainy days.”**

**Response:**

Many thanks for your suggestion. To improve the level of English writing, we have checked the full text of repetitive sentences and changed the expression of repetitive sentences. In addition, professional editing agencies have been invited to help improve English writing.

**2. Literature Review :Similar statements made by different authors have not been integrated, resulting in repetition of facts and a lack of cohesion and logical flow. Please rewrite sentences like “Moreover, the longer stopping sight distance needed due to a reduction in friction between the tiers and the wet road surface might be more challenging on curves.”**

**Response:**

Many thanks for pointing out this issue.We reorganized and compiled the Literature Review as follows.

Studies have demonstrated that the wet and slippery nature of roads, accompanied with the decrease in friction, caused by rainfall is a key variable in traffic accidents [1–5]. Evidently, with less friction, the braking distance of the vehicle increases at the same speed, which triggers frequent collisions in rainy weather. In their research, Brodsky et al. demonstrated that the accident rate on wet and slippery roads on rainy days was 2–3 times higher than that on dry roads on sunny days. Moreover, the longer stopping sight distance required due to a reduction in friction between tiers and the wet road surface might be more challenging on curves [3]. To further study the reasons for the reoccurring rain collision accidents caused by the reduction in the friction coefficient, researchers focused on the relationship between the friction coefficient and the water film depth, and reached the consensus that there was a direct correlation between the reduction in friction coefficient and the water film depth on road surface [6–8]. Studies inferred that there are primarily two methods of regression analysis and simulation adopted in studying the relationship between the friction coefficient and the water film depth. Using artificial rainfall, Luo et al. carried out dynamic friction coefficient test experiments under different water film depth and determined that the reduction in the friction coefficient is directly related to the water film depth of the road surface [6]. In addition, via indoor water spray experiments, Do et al. determined that the relationship between the friction coefficient and the water film depth (less than 1 mm) agrees with the Stribeck curve [7]. Unlike regression analysis, the finite element simulation model was established to predict the friction coefficient under different water film depths, and it was deduced that at the same driving speed, the friction coefficient decreased with the increase in the water film depth [8]. Although the research methods differ, they all arrived at a consistent conclusion: The thicker the water film, the lower the friction coefficient. Further, the hydroplaning phenomenon may occur, which adversely impacts driving safety under rainfall conditions [9–11].

Hence, a prediction model for the water film depth is crucial in determining the road friction coefficient and evaluating the impact of rainfall on traffic safety. However, various geometric features of road and facility drainage capacities, as well as different rainfall intensities, exert different influences on the water film depth. Luo et al. adopted the variance analysis and multi-factor treatment statistical methods and concluded that the calculation of water film depth is affected by rainfall (rainfall intensity and duration) and pavement characteristics (cross slope, longitudinal slope, pavement material properties, texture, and penetration) [12]. Regarding the aforementioned complex factors, several investigations on the water film depth have been conducted, such as regression analysis and artificial neural network [13–16]. Among them, the most widely adopted method is the water film depth regression model established via experiments. For example, based on the in-door rainfall simulation experiment, the UK Road Research Laboratory proposed the conventional RRL model, i.e., the rain water depth relative to drainage length, rainfall intensity (5-min duration), and slope [13]. Anderson modified the parameters of the RRL model and proposed a water film depth prediction model for a plane impermeable surface [14]. Unlike the RRL model, which only considers two pavement texture depths (1.8 mm and 2.4 mm), Gallaway developed a water film depth prediction model for the U.S. Department of Transportation, which considers nine pavement texture depths. The relationship between rainfall intensity, cross slope, surface texture, drainage-path length, and water film depth are determined through artificial rainfall experiments [15]. The RRL, Anderson, and Gallaway models are simple and widely used by other researchers for developing water film depth prediction models [17–18], regression model verification [19–20], water film depth prediction [21–23], etc.

**3.“we proposed a prediction model for water film depth in theory.” However, when I entered “prediction model for water film depth” in science direct, there are so much papers, please clarify the corrections and difference between your work and previous studies.**

**Response:**

Many thanks for pointing out this issue.

We have emphasized the corrections and difference with other models in the Introduction and Conclusion respectively.

(1) Introduction:

Compared with the RRL, Anderson, and Gallaway models, the theoretical model established in this study considers the geometric characteristics of the real road environment and the drainage capacity of drainage facilities; therefore, it can accurately reveal the main factors affecting the water film depth and describe the influence of the rainfall intensity, road geometric characteristics, and facility drainage capacities on the water film thickness. Hence, the proposed model overcomes the limitations of regression models.

(2) Conclusion:

Compared with the regression model based on a specific experimental data, the theoretical prediction model based on hydraulic theory and mathematical models demonstrated wider applicability. The factors affecting the water film depth in the regression model (slope, rainfall intensity, and drainage length) are considered, as well as the actual road alignment and drainage capacity of drainage facilities.

**4. Your models come from others (Ministry of communications of china, Design Specification for Highway Alignment, PRC Industry Standard JTG D20-2017). So please clarify your work and others, and why you choose the modes, which part is others and which is your contributions.**

**Response:**

Many thanks for pointing out this problem.We have carefully studied and made further modifications in the process of model establishment and derivation. The issue is explained as follows.

(1) Interpretation of normative references:

The criterion (Ministry of communications of china, Design Specification for Highway Alignment, PRC Industry Standard JTG D20-2017) only clarifies the vertical slope that is prone to water accumulation on the road surface and the value range of the composite slope. This paper establishes four road water accumulation models based on the range of slope values and the combination of cross and longitudinal slopes.

(2) Formula source:

This article uses a formula in the specification (Formula 11), which is the calculation formula for the shallow triangular discharge capacity formula of a single cross slope, and reference is marked in the text. The rest includes the use of AutoCAD to draw a schematic diagram of the road area water model, and establishing the calculation formula of water film depth.

Now the revised are listed partly as follows:

Page 7:

Even though the same volume of water is gathered, the different road geometry features and convergence directions of flow ensure different water film depths. According to the gradient values of cross and longitudinal slopes, the sections with poor drainage are divided into four types. Correspondingly, from the geometric perspective, four geometric models for road surface water are established by the AutoCAD software, as presented in Table 1. Road surface water accumulation and water film depth refer to the volume and height of water, respectively, in the geometric model.

Page 8:

The geometric models of water film depths with different road geometric characteristics are established based on different combinations of cross and longitudinal slopes. To obtain the volume and height of the geometric model, i.e., road surface water accumulation and water film depth, it is necessary to further derive the volume and water depth calculation formulas of different geometric models. Subsequently, the derivation process of the mathematical formula is described in detail.

Page 13:

The calculation of the displacement adopts the shallow triangular discharge capacity formula of a single cross slope specified by the Specifications for Drainage Design of Highway (JTG/T D33-2012) [25], and it is expressed as.

Page 14:

The real-time water film depth of the road surface during rainfall is the water depth of the cross section. Based on Equation 12, the water film depth model can be derived as.

Page 15:

In this case, the volume of road surface water is the difference in the actual displacement of rainfall drainage facilities.

**5.Please give explanations in Table 2. Parameter value. How did you get these data, from experiments or reference papers? Same problems in your work with other data.**

**Response:**

Many thanks for your question.

According to different data sources, we reorganized the results section of the paper.

The verification of the model in this study was categorized into two situations. One was the situation with drainage facilities and normal operation, where the model prediction results were verified by comparing them with results from the existing regression model. Through field experiments, data pertaining to rainfall intensity, catchment width, catchment length, and water film thickness were obtained, and the prediction accuracies of the theoretical and regression models were analyzed to verify the feasibility and accuracy of the theoretical prediction model. The detailed description and field test data are shown in S1 Text in the Supporting Information.

In the second situation, typical data of the variables in the model when the drainage facilities cannot be operated normally or do not have drainage facilities were selected, and the rationality of the model was analyzed qualitatively. Based on the design resources and field investigation of the Binlai Expressway in Shandong Province and the Hangshaotai Expressway in Zhejiang Province, the principles for determining the data used to analyze the models are elaborated.

The principles for determining the data are as follows:

(a) The length and width of the catchment should be determined in accordance with the actual resultant gradient of road. For a common four-lane expressway in China, there is a 11.25 m gap between the central isolation belt and the hard shoulder; hence, a catchment width of 11.25 m is adopted here.

(b) The distance between drainage facilities is generally set to be 25–50m. When there are many lanes on expressways and first-class highways, a smaller distance should be adopted. In addition, the drainage facilities should be distributed more densely at the bottom of the concave vertical curve. Therefore, the catchment length is selected as 25 m in this study.

(c) Considering that a small resultant road gradient facilitates water accumulation better, both cross and vertical slopes are taken as 2%.

(d) Regarding the common asphalt pavement, the roughness coefficient of the asphalt pavement used in this study is 0.013.

(e) Concerning the design data of the Shandong Binlai Expressway and Hangshaotai Expressway, the radius of the vertical curve is 15 000 m here.

(f) Rainfall intensity is assigned according to the grade of the short-time rainfall table, as presented in Table 6.

**6.This paper is to predict the depth of water film, your results are based on what data? How to evaluate the reliability of predict results？The results compared with models that you proposed, which is a bias.**

**Response:**

Thank you very much for your question.The model validation in the article has been reorganized and modified, and the three questions are explained below respectively.

(1) Data

The data source is divided into two parts: measured data and selected data. The measured data can be viewed through Supporting information. The principle of data selection has been added and elaborated in the text.

(2) The reliability of predict results

The verification of the model in this study was categorized into two situations.

One was the situation with drainage facilities and normal operation, where the model prediction results were verified by comparing them with results from the existing regression model. Through field experiments, data pertaining to rainfall intensity, catchment width, catchment length, and water film thickness were obtained, and the prediction accuracy of the theoretical and regression models was analyzed to verify the feasibility and accuracy of the theoretical prediction model.

In the second situation, typical data of the variables in the model when the drainage facilities cannot be operated normally or do not have drainage facilities were selected, and the rationality of the model was analyzed qualitatively.

(3) The results compared with models that our proposed

Extensive citation of the model has already been stated in the Introduction.

The RRL, Anderson, and Gallaway models are simple and widely used by other researchers for developing water film depth prediction models [17–18], regression model verification [19–20], water film depth prediction [21–23], etc.

The basis for the model selection are explained before verification.

Currently, the most widely used water film depth regression prediction models are the RRL, Anderson, and Gallaway models. The RRL and Gallaway models consider the state of road water flow as sheet flow, without considering the effects of drainage facilities. However, this study considers roads with drainage facilities, which are typical on highways in China. In this study, unlike the flooding state, the water flow is first converged under the effects of the water-retaining belt and curb, and then discharged from the outlet. The water film depth is the water depth in the water-retaining zone or curb. The Anderson model considers impervious pavements, and the theoretical model used in this study disregards the penetration of the pavement; therefore, the Anderson model was used for comparative analysis.

# To Reviewer #2:

Thank you very much for the detailed comments. We appreciate your time and help in reviewing our manuscript, and the insightful comments you provided that have helped significantly improve the quality of this study. We have revised the paper very carefully according to your suggestions, and detailed explanations of all the issues are as follows.

**Response:**

**For the first comment, you better add a typical background data in abstract. For the second comment, if the safety control measures is not put forword, it seems make this work less significant, or the author at least mentions it in future work**

**Response:**

Many thanks for pointing out this problem. Modifications have been made based on your suggestions.

(1) Typical background data

Water film depth is a key variable that affects traffic safety under rainfall. According to the Federal Highway Administration (FHWA), approximately 5700 people are killed and more than 544 700 people are injured in crashes on wet pavement annually.

References

FHWA, 2021. Road Weather Management Program [Access date: 25/4/2021]. <https://ops.fhwa.dot.gov/Weather/weather_events/rain_flooding.htm>

(2) The safety control measures have been added it in future work.

It is necessary to further analyze the sensitive factors that affect the thickness of the water film, which will help to propose solutions to reduce the impact of the water film thickness from the road design stage. The theoretical model ignores the influence of pavement characteristics, and the pavement material correction coefficient can be included in future studies. Moreover, the increase in the water film depth caused by rainfall will reduce the pavement friction coefficient, and further affect the lateral stability of the vehicle and the stopping sight distance. A practical speed limit plan is required to ensure vehicle safety.

# To Reviewer #3:

We appreciate your time and help in reviewing our manuscript. Thank you very much for your affirmation of our work in this paper and many thanks for your detailed comments. We have revised the paper very carefully according to your suggestion, and all the modified contents have been marked in red font in the updated manuscript.

**Response:**

**The author has addressed the comments well. But for the third comment, the author gave a full explanation, but I didn't find corresponding change in the conclusion of the paper.**

**Response:**

Many thanks for pointing out this issue. Modifications have been made based on your suggestions .

However, there are still some problems yet to be addressed. First, only the flow section with the single cross slope triangle was considered during the calculation for the water displacement of drainage facilities. The prediction model for the water film depth still needs to be verified for drainage facilities with poor capacities. In addition, considering that the timeliness of road maintenance contributes to the variation in the drainage capacity of facilities, field tests are required to investigate the performance evolution of the drainage capacity in future studies. It is necessary to further analyze the sensitive factors that affect the thickness of the water film, which will help to propose solutions to reduce the impact of the water film thickness from the road design stage. The theoretical model ignores the influence of pavement characteristics, and the pavement material correction coefficient can be included in future studies. Moreover, the increase in the water film depth caused by rainfall will reduce the pavement friction coefficient, and further affect the lateral stability of the vehicle and the stopping sight distance. A practical speed limit plan is required to ensure vehicle safety.
