# Supplementary material for: Predicting the water film depth: A model based on the geometric features of road and capacity of drainage facilities
Source: PLoS One. 2021 Jul 2;16(7):e0252767. doi: 10.1371/journal.pone.0252767 (PMC8253438; doi:10.1371/journal.pone.0252767)
Supplement: Attachment — Submitted filename: Response to Reviewers.doc [file pone.0252767.s004.doc]

# TO Reviewer #1:

We appreciate your time and help in reviewing our manuscript. Thank you very much for your affirmation of our work in this paper and many thanks for your detailed comments. We have revised the paper very carefully according to your suggestion, and all the modified contents have been marked in red font in the updated manuscript.

**Response:**

**1.Your English writing is improved. While the Abstract needs further improvement.**

**Response:**

Many thanks for your suggestion. We reorganized and compiled the Abstract as follows. In addition, professional editing agencies have been invited to help improve English writing.

The water film depth is a key variable that affects traffic safety under rainfall conditions. According to the Federal Highway Administration, approximately 5700 people are killed and more than 544 700 people are injured in crashes on wet pavements annually. While several studies have attempted to address water film depth issues by establishing prediction models, a few focused on the relationship among road geometric features, capacity of drainage facilities and water film depth. To ascertain the influence of the geometric features of road and facility drainage capacities on the water film depth, the road geometry features were first classified into four types, and the facility drainage capacities were considered from three aspects in this study. Furthermore, the concept of short-time rainfall grade was proposed according to the results of the field test. Finally, the theoretical prediction model for the water film depth was conceived, based on the geometric features of road and facility drainage capacities with different rainfall intensities. Compared with the traditional regression prediction models, the theoretical prediction model clearly shows the effects of the geometric features of road and facility drainage capacities. When the road drainage facilities have no drainage capacity, the water film depth increases rapidly with the rainfall intensity. This model can be used to predict the water film depth of road surfaces on rainy days, evaluate the effect of rainfall on the driving environment, and provide guidance for determining safety control measures on rainy days.

**2. The corrections and differences between your work and previous studies are not enough.**

**Response:**

Many thanks for pointing out this issue.We reorganized and emphasized the corrections and difference with previous studies in Abstract, Introduction and Discussion and Conclusion respectively as follows.

(1) Abstract

While several studies have attempted to address water film depth issues by establishing prediction models, a few focused on the relationship among road geometric features, capacity of drainage facilities and water film depth.

IntroductionClearly, the aforementioned regression models are in accordance with some specific test data in their experience. Their applications are limited to the types of parameters covered by the models, and when the parameter values are within the scope of the database used in their derivation. Owing to the changeable combination of road slope, superelevation, and alignment, facility drainage capacities and environment, the regression model may not accurately describe the influence of various factors on the pavement water film depth.

To address the limitations of the above approaches, this study intends to establish a prediction model for the water film depth, with a full theoretical foundation and extensive application scope to clearly demonstrate the effect of the main parameters. The theoretical model for predicting the water film depth under different rainfall intensities is established by considering four geometry features of roads and three kinds of facility drainage capacities to predict the relationship among the water film depth, the geometric characteristics of the road and the facility drainage capacities accurately.

Compared with the regression models, the theoretical model established in this study considers the geometric characteristics of the real road environment and the drainage capacity of drainage facilities; therefore, it can accurately reveal the main factors affecting the water film depth and describe the influence of the rainfall intensity, road geometric characteristics, and facility drainage capacities on the water film thickness. Hence, the proposed model on a more theotical basis overcomes the limitations of regression models.

(2) Discussion and Conclusion

Compared with the regression model based on a specific experimental data, the theoretical prediction model based on hydraulic theory and mathematical models demonstrated wider applicability. The factors affecting the water film depth in the regression model (slope, rainfall intensity, and drainage length) are considered, as well as the actual road alignment and drainage capacity of drainage facilities.

**3.The results should be compared with more previous studies.**

**Response:**

Many thanks for pointing out this issue. We have added more previous studies to compare with the predicted results of this study.

Presently, the existing water film depth regression prediction models mainly include RRL, Anderson, Gallaway and Ji models, as shown in Table 1.

**Table 1. Water Film Depth Prediction Models**

| Source | Equation Form | Variables |
| --- | --- | --- |
| RRL | $d=0.017\times(L\times I)^{0.47}\times S^{-0.2}$ | water film depth d  length of flow path L  rainfall intensity I  slope S  texture depth TD |
| Anderson | $d=0.15\times(L\times I)^{0.5}\times S^{-0.5}$ |  |
| Gallaway | $d=3.38\times10^{-3}\times TD^{0.11}\times L^{0.43}\times I^{0.59}\times S^{-0.42}-TD$ |  |
| Ji | $d=0.1258\times TD^{0.7261}\times L^{0.6715}\times I^{0.7786}\times S^{-0.3147}$ |  |

When the drainage facilities are in normal use, the water film depth prediction formula (13) in this study is compared with the existing regression models, as presented in Fig 1. The value of flow path length is more than 0 and The highway texture depth is generally required to be greater than 0.55mm. Where the length of flow path is 20m and the texture depth is 1 mm. To maintain the consistency of the slope values in the above regression model, the influence of the longitudinal slope on the resultant slope should be reduced. Where the longitudinal slope is 0.5%, and the cross slope is 2%. It can be approximated that the cross slope is equal to the resultant slope.

**Fig 1. Comparison of prediction values among different water film depth models**

It can be seen from the Fig 1 that when the drainage length and slope are constant, the increasing trend of water film depth in present work is similar to that of the RRL model. When the rainfall intensity is 20mm/h, the water film depth calculated by the model in this paper is close to the value calculated by RRL and Gallaway. When the rainfall intensity is greater than 20mm/h, the calculation results in this study are between the RRL and Gallaway model. The comparison results indicate that the theoretical model can be used to predict the water film depth of the road surface.
